# Post-transcriptional regulation of GABAB receptor and GIRK1 channels by Nogo receptor 1

**DOI:** 10.1186/1756-6606-6-30

**Published:** 2013-07-06

**Authors:** Rachana Murthy, Jeeyong Kim, Xiankui Sun, Roman J Giger, David J Fink, Marina Mata

**Affiliations:** 1 Department of Neurology, University of Michigan and VA Ann Arbor Healthcare System (Neurology and GRECC), 5027 BSRB 109 Zina Pitcher Place, Ann Arbor, MI 48109, USA; 2 Department of Cell and Developmental Biology, University of Michigan, Ann Arbor, MI 48109, USA

**Keywords:** Nogo receptor, GABA B receptor, Inwardly rectifying potassium channel (GIRK1), Synapse

## Abstract

**Background:**

Type B GABA receptors (GABA Rs) play a critical role in synaptic transmission. We carried out studies to determine whether neuronal cell surface expression of GABAB-Rs might be regulated by the Nogo receptor 1 (NgR1).

**Results:**

siRNA knock-down of NgR1 resulted in a selective increase of GABAB R1 and GABAB R2 protein without altering the expression of GABAA receptor or GAD65. The increase in GABAB receptor subunits was unaccompanied by a change in mRNA, but inhibition of mTOR by rapamycin blocked the increase in GABAB protein. NgR1 siRNA also caused an increase in G protein coupled inwardly rectifying potassium channel (GIRK1). The increase in GABAB receptor and GIRK1 channel proteins was in the plasma membrane, determined by cell surface biotinylation. In *NgR1* knockout mice, the amount of GABAB R2 and GIRK1 in hippocampus-derived synaptosomes was increased.

**Conclusions:**

Together these findings suggest that NgR1 mediated modulation of synaptic transmission may be accomplished, at least in part, through modulation of G protein coupled receptors and channels.

## Background

The balance between plasticity and stability of synaptic connections in neuronal networks is maintained by a dynamic process of biochemical and structural modifications. Synaptic changes are prominent during development, but the process persists in more subtle forms throughout the lifespan and can be observed in response to learning, as a result of injury and in aging [1-3]. A number of glial inhibitors of axonal regeneration originally identified in injury models, are also found in neurons of the uninjured CNS and localized to synaptic sites where they function to restrict synaptic plasticity. In particular, NgR1 and its ligand NogoA have been implicated in activity dependent refinement of neuronal synapses in the CNS; in the visual cortex, NgR1 and NogoA are important for the consolidation of synaptic connections established during the critical period [4].

In the hippocampus NgR1 restricts formation of excitatory synapses [5], limits activity dependent synaptic strength and regulates dendrite spine morphology [6], while dynamic regulation of NgR1 in the forebrain is required for consolidation of long lasting memory [7]. Similarly, NogoA restricts synaptic plasticity in the adult hippocampus where NogoA neutralization, shRNA knockdown or deletion of NogoA induced changes in dendritic structure of pyramidal neurons and resulted in increases in long term potentiation [8,9]. These electrical and structural changes correlate with an increase in NMDA and AMPA receptor subunits and the scaffolding protein PSD95 that we previously found to occur in hippocampal neurons dendritic spines by mammalian target of rapamycin (mTOR) mediated activation following deletion of NogoA or NgR1 [10].

Less is known about the role of NogoA or NgR1 in non-glutamatergic synaptic connections. In transgenic mice, overexpression of NogoA causes progressive loss of inhibitory Purkinje cell terminals in deep cerebellar nuclei and deficits in motor coordination [11], a loss that is attributed to decreased expression of synaptic scaffolding proteins. Here we report a novel function for NgR1 in hippocampal neurons. We found that knock down of NgR1 enhances levels of GABAB receptors and the downstream GIRK channel in the plasma membrane by a post-transcriptional mechanism that engages the rapamycin sensitive mTOR pathway. The effect appears to be selective, as NgR1 did not affect expression levels of the GABAA receptor or glutamic acid decarboxylase 65 (GAD65).

## Results

### Post-transcriptional regulation of GABAB receptor by NgR1

**Figure 1:**
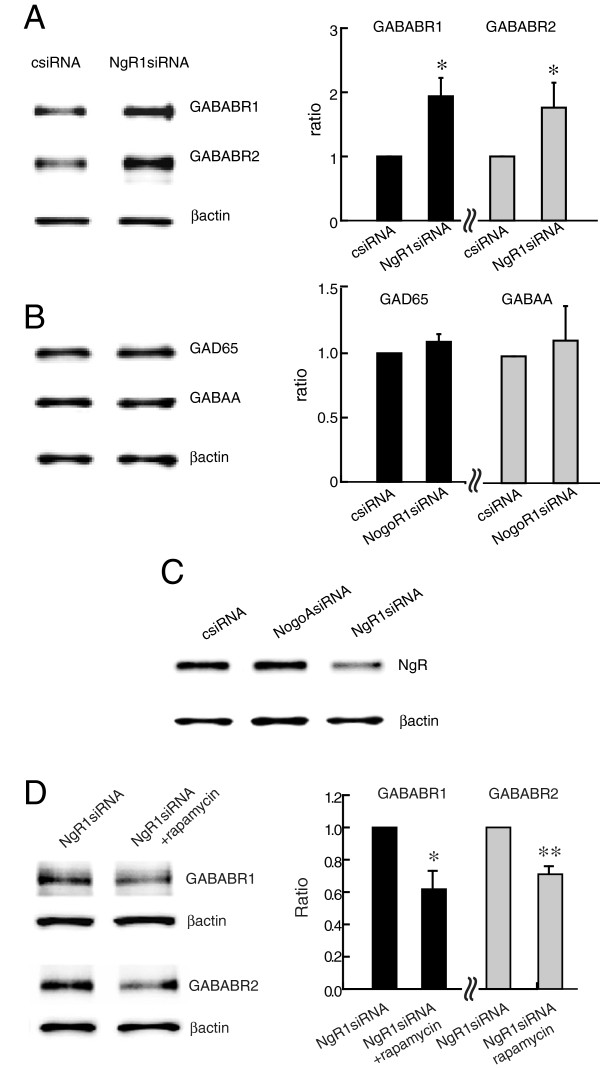
**Enhanced expression of GABAB receptor subunits by NgR1 siRNA is prevented by rapamycin.** Hippocampal neurons (DIV 14–17) treated with csiRNA or NgR1 siRNA. **A**. NgR1 siRNA significantly increased GABAB R1 and GABAB R2 protein levels. **B**. GABAA and GAD65 levels were unchanged. **C**. Knock down of NgR1 expression by NgR1 siRNA but not by csiRNA or NogoA siRNA used as controls. **D**. Rapamycin (100 nM) prevented the increased expression of GABAB R1 and GABAB R2 caused by NgR1 siRNA. Each sample was calculated as a ratio to β-actin and presented as percentage of control. * *P* < 0.05 and ** *P* < 0.01.

The NogoA-NgR1 interaction limits synaptic plasticity in part by restricting glutamate receptor expression. In order to know if NgR1 signaling may also influence surface expression of GABA receptors, we used primary postnatal hippocampal neurons (DIV14-17) treated with siRNA specific for NgR1 to determine levels of GAD65, responsible for GABA synthesis, and the GABA receptors GABAA and GABAB. Western blot analysis revealed that knock-down of NgR1 causes a significant increase in GABAB receptor subunits R1 and R2 protein compared to control siRNA (csiRNA) treated cultures (Figure 1A). Knock-down of NgR1 had no effect on expression levels of GABAA or GAD65 protein (Figure 1B). The knock-down of NgR1 by siRNA was confirmed by Western blot analysis (Figure 1C). mRNA levels measured by quantitative PCR analysis at the same time point did not change in NgR1 siRNA-treated hippocampal cultures compared to csiRNA-treated cultures (GABAB R1: csiRNA 1.01 ± 0.10 and NgR1siRNA 1.14 ± 0.11; GABAB R2: csiRNA 1.00 ± 0.03 and NgR1siRNA 1.01 ± 0.11). This indicates that the increase in GABAB R1 and GABAB R2 proteins represents a post-transcriptional process.

We have previously shown that siRNA knock-down of NogoA or NgR1 in hippocampal neurons increases mTOR phosphorylation and increases levels of glutamatergic receptors in dendritic spines; an effect that can be prevented by blocking mTOR signaling [10]. In order to determine whether mTOR activation caused by NgR1 knock down plays a similar role in the up-regulation of GABAB R1 and R2 subunit expression, we treated hippocampal neurons with rapamycin, an inhibitor of mTOR. Rapamycin blocked in part the increase in GABAB receptor subunits caused by NgR1 siRNA (Figure 1D) suggesting that GABAB receptor subunit expression may be under translational control downstream of mTOR. Rapamycin treatment of control hippocampal cultures produced no significant change in GABAB R1 or GABAB R2 protein levels (Additional file 1: Figure S1).

**Figure 2:**
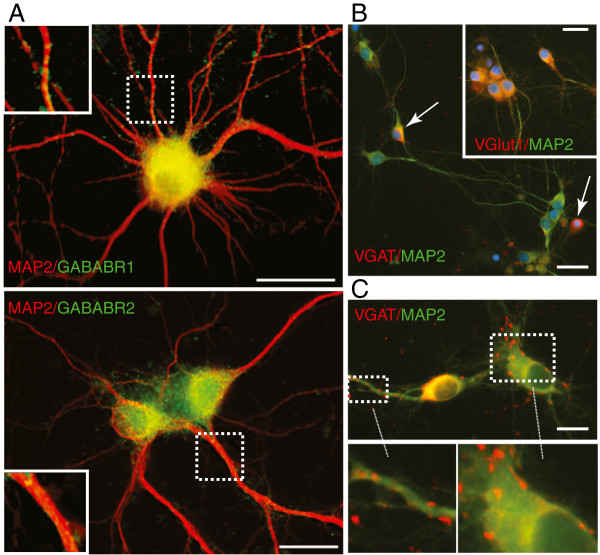
**GABAB R1 and GABAB R2 are localized on dendrites in the cultured neurons. A**. GABAB R1 (green) and GABAB R2 (green) localized in a punctate distribution along dendrites stained with MAP2 (red) in postnatal hippocampal neurons; scale bar = 25 μm. Inserts show higher magnification of the outlined area. **B**. Approximately 20% of the hippocampal neurons were GABAergic (arrows), stained with vGAT (red) and MAP2 (green), while the majority (80%) were glutamatergic stained with vGlut1 (red) and MAP2 (green) and shown in the insert. Scale bar = 25 μm. **C**. An extensive array of vGAT processes and terminals (red) contacting other non-GABA neurons (MAP2 green); scale bar = 10 μm.

GABAB receptors are G-protein coupled receptors (GPCRs) localized to the presynaptic and postsynaptic domains of excitatory and inhibitory neurons and mediate heterogeneous GABA responses. In the hippocampal cultures used in these experiments GABAB R1 and GABAB R2 were present in almost all the neurons, appearing as punctae on MAP2 positive dendrites (Figure 2A). The *in vitro* preparation consisted predominantly of glutamatergic neurons (75-80% as determined by vGlut1 staining) with the remainder (20-25%) characterized as GABAergic by vesicular GABA transporter (vGAT) immunostaining (Figure 2B). There was an extensive array of vGAT positive terminals on dendrites and soma of non-GABAergic neurons in these cultures (Figure 2C).

### NgR1 restricts GIRK1 levels

**Figure 3:**
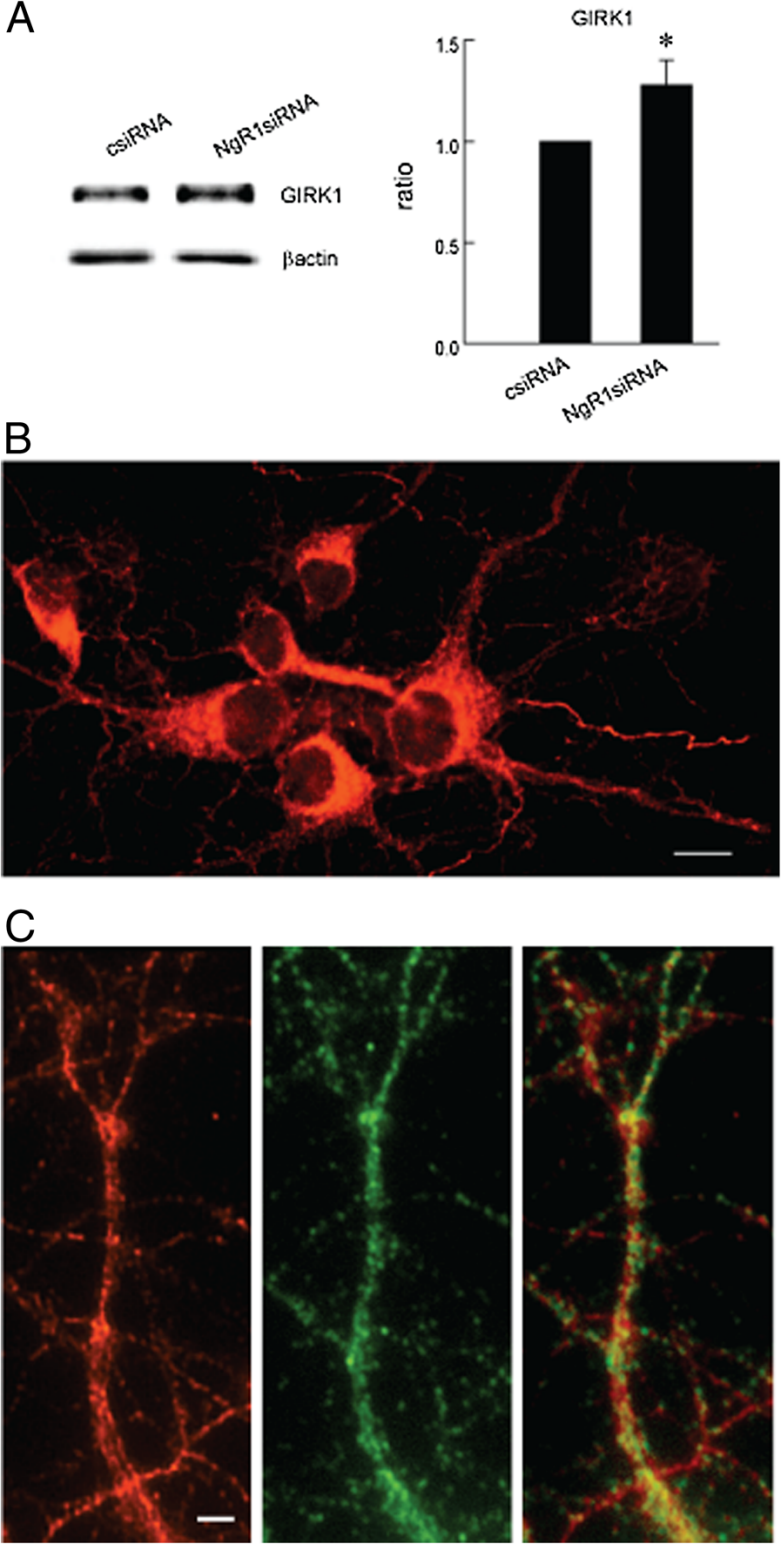
**Down-regulation of NgR1 increases GIRK channels. A**. Treatment of hippocampal neurons with NgR1 siRNA increases the level of GIRK1. Each sample was calculated as ratio to β-actin and presented as percentage of control. * *P* < 0.05. **B**. GIRK1 protein (red) is expressed in all neurons; scale bar = 10 μm. **C**. GIRK1 in dendrites (red) co-localized with GABAB R1 (green); scale bar = 2 μm.

G-protein coupled GABAB receptors influence second messenger systems and ion channels including the G-protein gated inwardly rectifying potassium channels (GIRKs) and voltage-dependent calcium channels, which together determine the slow and complex nature of the GABA response. GIRKs are tetrameric complexes of several channel subunits (GIRK1-4) and in the brain GIRK 1 associates primarily with GIRK2 and GIRK3. We chose to study GIRK1 because of its direct interaction with the GABAB R1 subunit and the special role it plays in determining channel activity [12,13]. We found that knock-down of NgR1 by siRNA in hippocampal neurons causes an increase in GIRK1 protein when compared to treatment with csiRNA (Figure 3A). GIRK1 immunostaining is seen in all hippocampal neuron cell bodies and along an extensive neurite network as shown in association with GABAB R1 (Figure 3B and C).

### The increase in GABAB subunits and GIRK occurs at the plasma membrane

**Figure 4:**
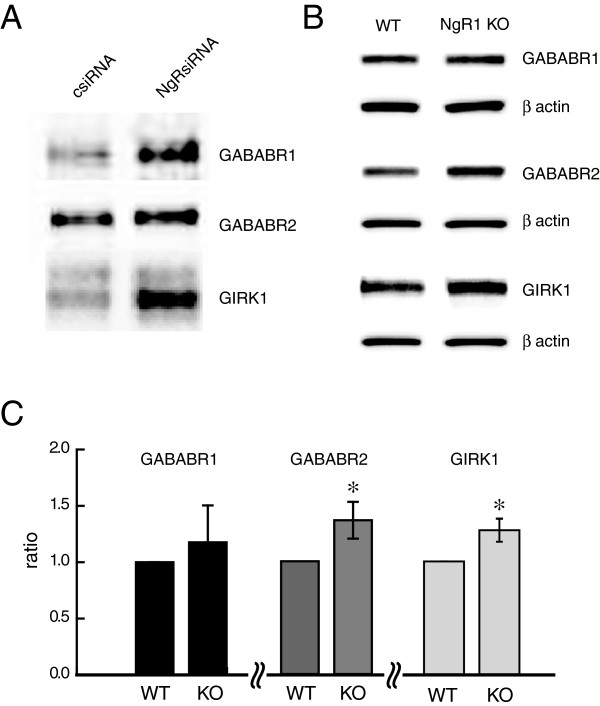
**Changes in GABAB and GIRK by down-regulation of NgR1 in plasma membrane and synaptosomes. A**. In vitro biotinylation of hippocampal neurons in culture shows increased GABAB R1, GABAB R2 and GIRK1 in the membrane fraction in cells treated with NgR1 siRNA. **B**. Increased GABAB R2, GIRK1 and to a lesser extent GABAB R1 were found in a synaptosomal preparation from hippocampal region from *NgR1* knockout mice. **C**. Quantitative analysis of results presented in B. Each sample was calculated as a ratio to β-actin and is presented as percentage of control. * *P* < 0.05.

In order to assess if the changes we observed in GABAB receptor subunits and GIRK1 caused by NgR1 siRNA reflect the levels of those proteins in the plasma membrane, we performed surface protein biotinylation of hippocampal neurons in culture treated with either NgR1 siRNA or csiRNA. After cell lysis, normalization and pull down of biotinylated proteins, equal amounts of sample were assayed from each experimental condition. Western blot analysis shows an increase in surface GABAB R1, GABAB R2 and GIRK1 in cultures treated with NgR1 siRNA as compared to csiRNA treated cells (Figure 4A). No contamination with cytosolic protein was observed as GAPDH was not seen by Western blot in these samples.

### GABAB and GIRK1 are increased in synaptosomes of *NgR1* knockout mice

To examine whether the changes induced by NgR1 siRNA in primary hippocampal neurons *in vitro* also occur *in vivo,* we isolated synaptic density fractions from hippocampal tissue of adult wildtype and *NgR1* knockout mice [6]. Analysis of protein levels in synaptosomal preparations from hippocampus of adult brains showed similar changes to those seen in hippocampal neurons *in vitro.* GABAB R2 and GIRK1 proteins were significantly increased in the synaptosomes from *NgR1* knock out as compared to control mice (Figure 4B), suggesting that the upregulation is occurring at synapses in vivo. While the GABAB R1 level also increased, this did not reach statistical significance when compared to control (Figure 4C).

## Discussion

We explored the role of NgR1 in modulating expression of GABA receptors in hippocampal neurons using siRNA knock-down and *NgR1* knockout mice. We found that NgR1 modulates levels of GABAB receptors and GIRK channel at the plasma membrane and in synaptosomes. The changes we found appear to be specific as NgR1 knock down does not modify the GABAA receptor or GAD65 protein levels. The regulation of GABAB expression by NgR1 is post-transcriptional and mediated by the rapamycin sensitive mTOR pathway, similar to the mechanism that we previously reported in the regulation of glutamate receptor expression by NogoA-NgR1 signaling, and that has been implicated in the development of LTP and dendritic spine morphology [8-10,14].

GABAB receptors are heterodimers composed of GABAB R1 and GABAB R2 subunits and in the hippocampus both subunits are present in dendrites where they localize to the extra-synaptic membrane of spines and dendritic shafts where they mediate the slow inhibitory postsynaptic currents [15,16]. Heterodimerization of the receptor is a requisite for stable surface expression of GABAB receptors [17] and the density of membrane-localized receptors is one factor in determining signaling strength in response to changing physiological conditions. In our cultured hippocampal neurons GABAB R1 and R2 appeared as puncta on dendrites and cell bodies of glutamatergic and GABAergic neurons, and NgR1 knockdown significantly increased the amount of the GABAB receptor subunits in the plasma membrane without altering mRNA levels. These results suggest that conditions altering NgR1 activation may have effects on GABA mediated signaling.

GABAB receptors traffic through the ER and Golgi networks for delivery to the plasma membrane and once at the cell surface undergo constitutive endocytosis and are rapidly recycled to the cell surface [18,19]. However in hippocampal neurons GABAB R1 and R2 subunits may also heterodimerize and assemble to form functional receptors at the plasma membrane, a process that is highly dynamic and regulated by intra- and extra-cellular cues [20]. Our studies indicate that rapamycin-sensitive mTOR signaling mediates the up-regulation of GABAB receptor expression, thus providing a post-transcriptional mechanism by which the neuron may exert local control of GABAB receptor expression in response to changing NgR1 levels.

While GABAB R1 contains the ligand binding domain, GABAB R2 associates with pertussis toxin sensitive G family of proteins (Gαi/o). Activation of the receptor triggers GTP-dependent release of G-protein heterodimers which regulate second messengers and ion channels. Oligomerization of GABAB receptors and GIRK channels produces stable macromolecular complexes that localize to the plasma membrane [21] where GIRK1 appears to interact in a direct and specific manner with GABAB R1 [12]. Our results are consistent with a physical or functional interaction of GABAB and GIRK1, as levels in the cell membrane and in synaptosomes appeared jointly regulated by NgR1. Our work does not address the functional outcome of GABAB and GIRK regulation by NgR1, but GABAB and GIRK complexes are known to generate slow inhibitory post-synaptic responses and to reduce network activity [22,23].

## Conclusions

We found that GIRK1 levels are regulated together with GABAB receptor subunits B1 and B2 in the plasma membrane and in synaptosomes, suggesting that NgR1 signaling may contribute to synaptic modifications by restricting GABAB-GIRK complex mediated effects in the hippocampus. Taken together, these data suggest that NogoA-NgR1 signaling may play a modulatory role in the complex regulation of neuronal excitability and/or synaptic network activity.

## Methods

### Tissue culture

Hippocampal neurons were isolated from postnatal day 2 (P2) Sprague Dawley rats (Charles River, Portage, MI, USA) and were cultured in defined Neurobasal medium (Invitrogen, Carlsbad, CA, USA) as previously described [10]. The studies were approved by the VA Ann Arbor Healthcare System Animal Studies Committee, and appropriate measures were taken to minimize pain and discomfort. Tissue culture studies were performed at DIV14-17 when the neurons display a mature phenotype. Rapamycin (Tocris Bioscience, Bristol, UK) was added to the culture medium for 24 hours as indicated.

### siRNA preparation and transfection

ON-TARGET plus SMARTpool siRNA directed against NgR1 and ON-TARGET plus siCONTROL non-targeting pool siRNA were used (Dharmacon, Chicago, IL, USA). The siRNA sequences were as we described previously [10]. Transfection solution contained 2.5 μl of siRNA in 47.5 μl of antibiotic free culture medium and 2 μl of DharmaFECT siRNA transfection reagent 3 (Dharmacon) in 48 μl of cultured medium. The mixture was incubated for 20 min at 21°C. Hippocampal neurons (DIV14) were treated with the transfection solution for 72 h, at which point cell homogenates were collected for RNA and protein analysis.

### RNA isolation and quantitative PCR

Total RNA was isolated using the RNeasy Mini kit (Qiagen, Valencia, CA, USA) from hippocampal neurons transfected with either NgR1siRNA and csiRNA as described above. RNA quality was monitored by agarose electrophoresis. Reverse transcription was conducted using the Superscript Reverse Transcriptase II kit (Invitrogen) and the cDNA obtained used for quantitative PCR (qPCR) determination. Primers were mixed with iQ5 SybrGreen Supermix (BioRad Laboratories, Hercules, CA, USA) and amplification was carried out in 25 μl of iQ5 Real-Time PCR detection system using a two-step qPCR protocol with the initial denaturing step at 95°C for 3 minutes followed by 40 cycles (95°C for 20 seconds, 58°C for 30 seconds). The primers used for qPCR are as follows. For GABAB R1 amplification, the forward primer is 5′-ACAAGGGGCTGCTGCTGCTG −3′ and the reverse prime is 5′-GATAGCCATGCCCACGGCCC −3′. For GABAB R2 amplification, the forward primer is 5′-AAACGTTCAGGCGTGGGGCC-3′ and the reverse primer is 5′-CCGCTGGTGCCTGCTACTGG-3′. For β-actin amplification, the forward primer is 5′-CAGTTCGCCATGGATGACGATATC-3′, and the reverse primer is 5′-CACGCTCGGTCAGGATCTTCATG-3′. Levels of mRNA for GABAB R1 and R2 were calculated in relation to levels of β-Actin mRNA (internal control) using the 2^−ΔΔCt^ method [24].

### Western blot

Primary hippocampal neurons were collected in lysis buffer (50 mM Tris, 10 mM NaCl, 1% Nonidet P-40, 0.02% NaN3) containing 1% protease and phosphatase inhibitor cocktail (Sigma, St Louis, MO, USA) and Western blot performed and analyzed as described [10]. Primary antibodies were: anti-NgR1 (1:100, R&D Systems, Minneapolis, MN, USA), anti-GABAB R1 (1:200, Santa Cruz Biotechnology, Santa Cruz, CA, USA); anti-GABAB R2 (1:400, NeuroMab, UC Davis/NIH, CA, USA) anti-GIRK1 (1:200, Sigma), anti-GABAA α1 subunit (Millipore, Billerica, MA, USA), anti-GAD65 (1:1000, Chemicon, Temecula, CA, USA) and anti β-actin (1:4000, Sigma). Secondary antibodies were peroxidase coupled (1:2000, Santa Cruz Biotechnology or 1:3000, Jackson ImmunoResearch, West Grove, PA) and amplified using SuperSignal chemoluminescence reagent (Pierce). Chemiluminescence of each protein band was quantitated using a ChemiDoc (Bio-Rad) device. The quantitative analysis was performed as follows. The chemiluminescent intensity of the band representing the protein of interest (with the background subtracted) was compared to the chemiluminescent intensity of the corresponding internal control β-actin (background subtracted) for each sample in the experimental group, and then the results were compared to the control samples from the same blot quantitated in a similar manner with the control set as 1, so as to determine the percentage change between the experimental treatment and the control treatment for each sample group. The results presented represent a summary of at least 3 independent experiments.

### Cell surface biotinylation

Following siRNA transfection, hippocampal neurons underwent cell surface biotinylation using the Pierce Cell Surface Protein Isolation kit (Thermo Scientific, Rockford, IL, USA). Cells were labeled with the non-cell membrane permeabilizing reagent EZ-Link Sulfo-NHS-Biotin for 30 min at 4°C and the labeling reaction was stopped by adding the Quenching solution according to the kit instructions. At the completion of the reaction cells were washed and lysed in the presence of protease inhibitors at 4°C. Cell lysates were centrifuged at 10,000 × g for 2 minutes and the supernatants collected were normalized to have similar amounts of total protein. The biotinylated proteins from each sample were isolated using NeutrAvidin Agarose. The bound proteins were washed and released by incubating in SDS-PAGE sample buffer containing 50 mM DTT. Equal amounts of treated and control samples were used for SDS-PAGE and Western blot analysis. Anti-GAPDH (Santa Cruz Biotechnology) representing the cytosolic compartment, was used as control for the purity of the sample and detected no protein band.

### Immunocytochemistry

Hippocampal neurons were fixed, blocked and probed overnight with the following antibodies anti-GABAB R1 (1:200, Santa Cruz Biotechnology); GABAB R2 (1:400, NeuroMab) anti-GIRK1 (Sigma), anti-MAP2 (1:1000, Millipore, Billerica, MA, USA), anti-vGlut1 (1:1000, Chemicon International) and anti-vGAT (1:1000, Millipore). The secondary antibodies were tagged to Alexa Fluor 594 or 488 (1:2000, Invitrogen). Images were acquired using a Nikon E1000 fluorescent microscope with a Photometric Cool Snap ES camera and Metamorph software.

### Preparation of synaptosomes

Synaptosomes were obtained from 7–8 week old male wild type (*NgR1+/+*) and *NgR1* (*NgR1*−/−) knockout mice (C57Bl/6 background) that were generously provided by Marc Tessier-Lavigne (Rockefeller University, NYC, NY) [25]. The isolation of synaptosomes was performed as described previously [14]. Briefly, each preparation was generated from hippocampi of four *NgR1*knockout mice or four wild-type mice and the tissues were homogenized in a 0.32M sucrose solution containing 0.1 mM CaCl_2_, 1 mM MgCl_2_, 0.1 mM PMSF, 25 mM NaF, and 1 mM Na_3_VO_4_. The sucrose concentration was adjusted to 1.25M, using a 2M sucrose solution containing 0.1 mM CaCl_2_, and synaptosomes were collected from the 1.25M/1M interface of a prepared sucrose gradient by centrifugation at 100,000 × g for 3 hr at 4C in a Sorvall Ultracentrifuge using a SW41 rotor. Isolated synaptosomes were adjusted to the same final protein concentration and analyzed by SDS-PAGE. Three synaptosomal preparations were obtained from *NgR1* knock-out mice and three similar samples were obtained from control mice.

### Data analysis

Data are shown as mean ± SE. The statistical significance of the difference between treated and control group was determined by one way ANOVA using SPSS 12.0 for Windows (SPSS Inc. Amonk, NY, USA) with p < 0.05 considered significant. All experiments were performed at least three times using independent samples.

## Abbreviations

AMPA: 2-amino-3-(3-hydroxy-5-methyl-isoxazol-4-yl)propanoic acid; Ca: Calcium; cDNA: Complementary DNA; CNS: Central nervous system; csiRNA: Control siRNA; DIV14-17: Primary postnatal hippocampal neurons; DNA: Deoxyribonucleic acid; GABA: Gamma aminobutyric acid; GABAB-R: Type B GABA receptor; GAD65: Glutamic acid decarboxylase 65; GAPDH: Glyceraldehyde phosphate dehydrogenase; GIRK: G protein-coupled inwardly-rectifying potassium; GPCR: G-protein coupled receptor; LTP: Long term potentiation; MAP2: Microtubule-associated protein 2; mRNA: Messenger ribonucleic acid; mTOR: Mammalian target of rapamycin; NgR1: Nogo receptor 1; NMDA: N-methyl-D-aspartic acid; NogoA: Neurite outgrowth inhibitor A; PSD95: Postsynaptic density protein 95; QPCR: Quantitative PCR; siRNA: Small interfering RNA; VGAT: Vesicular GABA transporter; VGLUT: Vesicular excitatory amino acid transporter.

## Competing interests

The authors declare no competing financial interests.

## Authors’ contributions

JK: Performed research and analyzed data; XS: Performed research; RM: Performed research and analyzed data; RJG: Performed research, analyzed data and wrote the paper; DJF: Analyzed data and wrote the paper; MM: Designed the research, analyzed data and wrote the paper. All authors read and approved the final manuscript.

## Supplementary Material

Additional file 1: Figure S1 Treatment of hippocampal neurons with 100 nM repamycin for 24 h did not change baseline levels of GABAB R1, GABA R2, GABAA or GIRK1 protein, calculated as a ratio to the corresponding β-actin internal control. Click here for file
